# Supplementary material for: Characteristics of the Colorless Polyimide-Based Flexible X-ray Detector with Non-Fullerene Acceptor Polymer
Source: Nanomaterials (Basel). 2022 Mar 10;12(6):918. doi: 10.3390/nano12060918 (PMC8948920; doi:10.3390/nano12060918)
Supplement: Supplementary file 1 [file nanomaterials-12-00918-s001.zip › nanomaterials-1581046-supplementary.pdf]

nanomaterials

MDPI

Supplementary Materials

Characteristics of the Colorless Polyimide-Based Flexible X-ray Detector with Non-Fullerene Acceptor Polymer

Jehoon Lee [1], Jongkyu Won [1], Duhee Lee [1], Hailiang Liu [1] and Jungwon Kang [1,2,*]

1 Department of Electronics and Electrical Engineering, Dankook University, Yongin-si 16890, Korea; usyj0512@gmail.com (J.L.); 1jongkyu@naver.com (J.W.); clemenslee94@gmail.com (D.L.); liuhailiang107@gmail.com (H.L.)
2 Convergence Semiconductor Research Center, Dankook University, Yongin-si 16890, Korea
* Correspondence: jkang@dankook.ac.kr

1. Detector Characteristic Hysteresis According to X-ray Irradiation

The hysteresis was measured before and after X-ray exposure to the detector using PBDB-T:$PC_{71}BM$ and PBDB-T:ITIC active layer. The J-V characteristics under dark condition were evaluated according to the before X-ray exposure and after X-ray exposure time interval (0, 2, 4 min), and it was shown in Figure S1. The X-ray was irradiated under the conditions of 80 kVp tube volatage, 60 mAs tube current, and 1.57 second irradiation time.

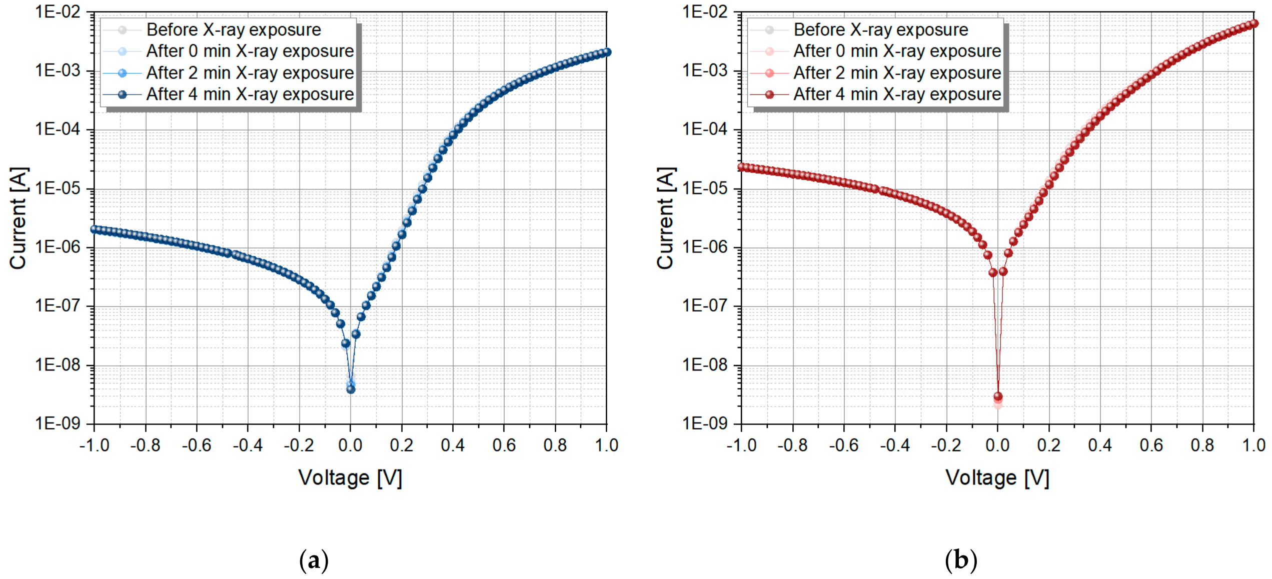

Figure S1. J-V characteristic hysteresis measured at dark conditions, before and after X-ray irradiation to the detector using (a) PBDB-T:$PC_{71}BM$ and (b) PBDB-T:ITIC active layer.

2. Comparison Experiment of Rigid and Flexible CsI(Tl) Scintillator

To fabricate a flexible X-ray detector, both the device and the scintillator must have flexible properties. Therefore, rigid and flexible scintillators were evaluated by coupling to the photodetector with PBDB-T:ITIC active layer. Figure S2 (a) shows the rigid type (Hamamatsu J13112) and flexible type (Hamamatsu J1477) CsI(Tl) scintillator emission spectrum graph when the X-ray is irradiated. The flexible scintillator shows higher emission than the rigid type. Figure S2 (b) shows the CCD and sensitivity when the two different types of scintillators were coupled. The CCD and sensitivity were improved, due to the flexible types having better emission than the rigid types.

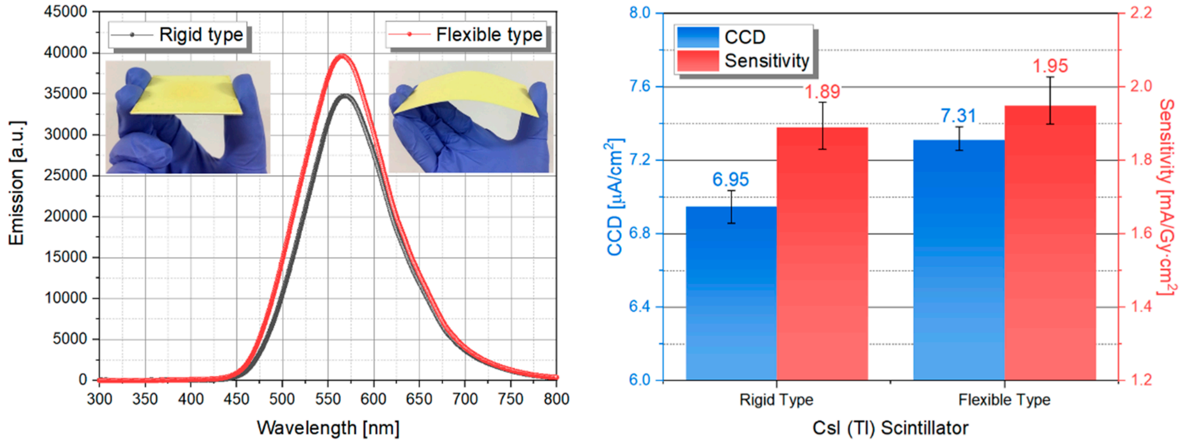

(**a**) (**b**)

Figure S2. (a) Emission spectrum of the rigid and flexible CsI (Tl) scintillator under the same X-ray condition, and (b) the CCD and sensitivity of the X-ray detector with different scintillators.

3. The Performance Comparison of Flexible X-ray Detector

Table S1. The performance comparison of flexible X-ray detectors.

Detection method	Materials	Sensitivity [mC/Gy·cm²]	Tube voltage [kVp]	Scintillator	References
Indirect-type Flexible detector	PBDB-T:ITIC	3.003	80	CsI(Tl)	This work
	P3HT:O-IDTBR	3.08	80	CsI(Tl)	[1]
	$MAPbI_3$	0.6-1.27	30-120	CsPbBr3	[2]
Direct-type Flexible detector	Ni-DABDT	0.986	26	-	[3]
	Cd_2TeI_6	0.0763	20	-	[4]
	PDMS:Bi_2O_3	0.2036	60	-	[5]
	$MAPbI_3$	2.527	70	-	[6]

References

1. Lee, J.; Kang, J. Characteristics of a Flexible Radiation Detector Fabricated with Non-Fullerene Acceptor for an Indirect-type X-ray Imaging. *J. Instrum.* **2019**, 14, C03008.
2. Heo, J.H.; Park, J.K.; Yang, Y.; Lee, D.S.; Im, S.H. Self-powered flexible all-perovskite X-ray detectors with high sensitivity and fast response. *iSci.* **2021**, 24, 102927.
3. Li, Z.; Chang, S.; Hu, Y.; Huang, Y.; Au, L.; Ren, S. Flexible Lead-Free X-ray Detector from Metal-Organic Frameworks. *Nano Lett.* **2021**, 21, pp.6983-6989.
4. Guo, J.; Xu, Y.; Yang, W.; Xiao, B.; Sun, Q.; Zhang, X.; Zhang, B.; Zhu, M.; Jie, W. High-Stability Flexible X-ray Detectors Based on Lead-Free Halide Perovskite Cs_2TeI_6 Films. *ACS Appl. Mater. Interfaces* **2021,** 13, pp.23928-23935.
5. Mao, L.; Li, Y.; Chen, H.; Yu, L.**;** Zhang, J. A high-sensitivity flexible direct x-ray detector based on bi2o3/pdms nanocomposite thin film. *Nanomaterials* **2021,** 11.
6. Shrestha, S.; Fischer, R.; Matt, G.J.; Feldner, P.; Michel, T.; Osvet, A.; Levchuk, I.; Merle, B.; Merle, B.; Chen, H.; Tedde, S.F.; Schmidt, O.; Hock, R.; Rührig, M.; Göken, M.; Heiss, W.; Anton, G.; Brabec, C.J. High-performance direct conversion X-ray detectors based on sintered hybrid lead triiodide perovskite wafers. *Nat. Photonic.* **2017**, 11, pp. 436-440.
